# Health sciences students' and instructors' perceptions of the emergency switch to virtual internship amid the COVID-19 pandemic: A case from Qatar

**DOI:** 10.3389/fmed.2022.939416

**Published:** 2022-08-09

**Authors:** Hiba Bawadi, Hanan Abdul Rahim, Joyce Moawad, Rula Shami, Xiangyun Du, Alla El-Awaisi, Ayad Moslih Ibrahim Al-Moslih, Mohammad Diab, Ghadir Fakhri Al-Jayyousi

**Affiliations:** ^1^College of Health Sciences, QU Health, Qatar University, Doha, Qatar; ^2^College of Education, QU Health, Qatar University, Doha, Qatar; ^3^College of Pharmacy, QU Health, Qatar University, Doha, Qatar; ^4^College of Medicine, QU Health, Qatar University, Doha, Qatar

**Keywords:** students, readiness to emergency change, virtual internship, health sciences, COVID-19 pandemic, Qatar

## Abstract

In efforts to contain the COVID-19 pandemic, health colleges at Qatar University shifted their clinical training to virtual internships (VI) and project-based learning (PBL). The shift was new to students and faculty alike, and a major change that posed many challenges. This study aimed to explore the experience of changing to VIs during the pandemic from both the clinical instructors' and health sciences students' perspectives. A qualitative study was conducted based on the framework of readiness to change. It involved focus group discussions with students from the departments of Public Health and Human Nutrition and in-depth interviews with clinical instructors using appropriate online platforms. A total of 4 focus groups with 20 students and 4 interviews with instructors were conducted. Transcripts were analyzed following the inductive-deductive approach. The major themes that emerged from the analysis described students' and clinical instructors' perceptions of the necessity and efficiency of the switch to VI; the design of the VI and the extent of the clinical/field experience and skills that it offered; confidence in the ability to succeed in this type of internship and confidence about reaching expected goals; academic and moral support from clinical faculty and coordinators and the communication process with faculty and preceptors; and finally, the benefits gained and how employers would view this type of internship. Health sciences students' readiness for VI was generally low. Several student and faculty needs have to be addressed, specifically regarding the design of the program and the level of preceptors' communication with students. The findings would direct health programs, clinical instructors, and preceptors to better understand students' needs and efficiently plan for virtual internships during not only emergencies but also whenever there is a need to deliver online experiential learning courses.

## Introduction

Worldwide, many educational institutions have experienced an urgent shift of teaching and learning activities to full-scale online modes in response to the COVID-19 pandemic. Although there are benefits to online education, including learning flexibility and inclusiveness, there are several challenges: increased workload for instructors, difficulty motivating students, lack of opportunities for interaction, technical issues, and lack of appropriate teaching materials (1).

Practice-based learning is the backbone of effective learning across all health and medical professions (2). Early practical experience in medical education strengthens learning and is associated with an important set of learning outcomes. Such outcomes of early clinical learning include increased student satisfaction, adaptation to clinical environments, reducing stress and enhancing confidence upon interacting with patients, learning self-reflection and appraisal skills, developing professional identity (3), and building the set of essential competencies for medical students (collaboration, communication, problem-solving integrity, responsibility, and respect) (4–7). While the above-mentioned literature outlines the benefits and challenges of virtual health education, the studies were mostly conducted in well-planned programs where students participated by choice and with the availability of alternative or supplemental modes of learning. Some recent studies explored medical students' perceptions regarding the effectiveness of online learning and the learning challenges faced during the COVID-19 pandemic (8, 9). For example, in a study from The United States of America (USA) (10), and another from The Kingdom of Saudi Arabia (KSA) (8), the majority of medical students expressed that the clinical aspect was a main missing component of VI. In Brazil, there was diversity among medical students' responses regarding their willingness to maintain their regular clinical training during the pandemic, but more than 80% of their expressed views reflected widespread insecurity toward the current situation (11). However, in a study from Singapore, up to two-thirds of medical students expressed a need to go back to their regular on-site training during the COVID-19 pandemic (12).

Qatar University (QU) health programs, like many of their counterparts around the world, had been challenged by the COVID-19 pandemic. At the College of Health Sciences (CHS), the Department of Public Health (PH) and Department of Human Nutrition (HN) faculty and clinical preceptors struggled to create safe and appropriate alternatives to practice-based learning due to the suspension of routine services in the health care system. Similarly, students struggled to cope with the abrupt change in their mode of learning. These two programs are only offered for female students at QU.

When the university started the virtual clinical practice, the PH department shifted its health education practicum to a project-based internship (PBI) replacing their clinical training in Primary Healthcare Centers in which they would finalize their internship after having their community-level training at the Ministry of Public Health and other public health organizations in Qatar. In the PBI, students constructed different instruments after reviewing the literature to assess the needs of specific audiences in regard to different public health topics. They collected online data and conducted the analysis. Then, they tailored the findings toward specific activities to promote the health of the chosen audiences or to enhance the services provided for them in various settings. Students also built creative models to plan for implementing these activities and services and suggested a variety of methods to monitor the implementation process to enhance their outputs. In addition, they planned for effect evaluation by suggesting specific outcome indicators and different research designs.

The HN internship is a structured program including rotation in community sites such as Qatar Diabetes Association (QDA) and clinical sites such as cardiac, oncology, renal, food service, and other medical departments of Hamad Medical Corporation (HMC) hospitals. During the pandemic, most clinical rotations were immediately shifted to virtual rotations, and different student tasks were suspended such as patient education, counseling, and working in interdisciplinary teams. These were replaced by an extended number of hours of professional development classes where students attended some workshops by dietitians abroad and got more involved in case simulations using the telehealth concept, case studies, case presentations, preparation of awareness campaigns, and writing articles.

### Theoretical framework

The decision to shift to full-scale online education was taken by the national authority overseeing the pandemic response. Under such emergency circumstances, students and instructors were not prepared for the transition, which may be critical for their readiness for change. In turn, readiness for change is important for the success of targeted outcomes (13, 14). Originating in organizational studies, readiness can be defined as “the cognitive precursor to the behaviors of either resistance to, or support for, a change effort” (15), or “recipients' beliefs regarding the appropriateness of, support for, and value of the change (16). As such, it is critical to understand the students' perceptions and experiences in this unique setting.

Holt et al. (17) suggest that readiness for change is a comprehensive attitude influenced simultaneously by a number of factors, including the content (i.e., what is being changed), process (i.e., how the change is being implemented), context (i.e., circumstances under which the change is occurring), and individuals involved (i.e., characteristics of those being asked to change). It is also influenced by how the change recipients, as an individual or as a group, are cognitively and emotionally prone to accept, adapt and cooperate with a particular plan to change the situation.

In line with the framework of readiness for change to facilitate effective VIs, it is essential to support students with initial readiness for emergency change, addressing the following five aspects. First, discrepancy refers to understanding the need to use VIs in such an emergency in order to reach the aspired goals, and have the knowledge/skills about VIs. Second, appropriateness refers to students' belief that the current use of the online learning platform and the design of VIs is appropriate for the given purpose. When students understand how the change is implemented and what strategies are being used to support VIs, they have a better chance to develop a strong belief in the appropriateness of the change and get motivated in and engaged with the changed mode of learning (18–20). Third, efficacy, which refers to students' confidence in their own and the institution's ability to conduct online training for the aspired goals, is an essential aspect for change readiness. Understanding students' efficacy is important to develop educational strategies to prepare students with the necessary knowledge and skills regarding online learning tools and platforms when shifting from face-to-face to the online environment (21).

A fourth aspect of readiness for change is principal support. Leadership support is significant to improving motivation and self-efficacy to ensure the success of change efforts (22). Literature on online education also suggests that it is essential to establish a supportive learning environment to facilitate online active learning and student-centeredness (23–25). Finally, valence is a significant aspect of readiness to change, which refers to the perception of long-term personal benefit for the learner in the VI experience. Seeing long-term benefits can help change recipients to develop motivation, efficacy, and strategies in coping with challenges in the change process (7, 14).

While current literature highlights the critical role of readiness for change, little is known about how change recipients report their readiness in the context of an urgent shift, when the decision to make the change is enforced externally. Understanding CHS students' experiences while shifting to virtual internships, and the factors shaping this process would direct health programs, clinical instructors, and preceptors to better understand students' needs and efficiently plan for virtual internships during a not only emergency but also whenever there is a need to deliver on-line experiential learning courses. This study aimed to explore health sciences students' and instructors' experience of an emergent shift to virtual internship and their perceptions of VI replacing on-site experiential learning.

## Methods

A qualitative design was employed in this study, which took place at the time when the COVID-19 pandemic was at its peak in Qatar (Spring semester 2020). Purposive sampling was followed to recruit participants who are considered rich cases and would help answer the overarching research question. Our study ran in two parallel streams, one involving focus group discussions with senior female students registered for clinical practice from the departments of Public Health (PH) and Human Nutrition (HN), and the second involved conducting in-depth interviews with clinical instructors (clinical coordinators, clinical faculty, and preceptors). Online platforms (WebEx and Microsoft teams) were used for data collection, given regulations surrounding social distancing and restrictions on in-person gatherings. An interview guide and a focus group guide were developed considering the main constructs of the readiness framework and based on the discussions of the research team and a review of the relevant literature. Interviews and focus groups were recorded with participants' permission, and a member of the research group took written notes simultaneously.

### First stream: Students

To invite participants, emails and reminders were sent out through Blackboard to all students registered for clinical training in spring 2020, providing research information and a link to an electronic consent form. We conducted four focus groups until data saturation was reached, where no new themes emerged (26). A trained member of the research team who is not involved in coordinating or delivering clinical training for the students facilitated the groups. The focus groups, conducted in English, were conducted online, each lasting 60–70 min.

### Second stream: Clinical coordinators and clinical faculty preceptors

Similarly, clinical instructors, including clinical coordinators and faculty responsible for bedside teaching in clinical sites were contacted by email with electronic consent. Four semi-structured interviews were conducted on online, each lasting 45 min.

### Data analysis

All interviews and focus groups were audio-recorded and transcribed into texts for analysis. An inductive-deductive approach was employed in the analysis. Inductive qualitative analyses were used to discover emerging themes, and the transcripts were analyzed for predetermined themes mapped to the readiness framework as well (27). Coding was the first step in the analysis process and it started after the first interview. A codebook was constructed and themes were added to the codebook as they emerged from each transcript. Constant comparisons were conducted to differentiate one theme from another and to identify the dimensions of each theme (28). With each addition of new data, themes were added and modified as needed. Finally, the themes were combined into a coherent description of the phenomenon. Two members of the research team worked independently to analyze the data. A different research team member who was not involved in the focus group discussion nor in conducting the individual interviews reviewed the transcribed data. The three members read the transcripts and identified the common themes separately then discussed the results and reached a consensus regarding themes and categories.

## Results

In total, we conducted four focus groups with students from CHS (*N* = 20), two with senior health education students from PH (*N* = 11), and two with senior students from HN (*N* = 9). We also conducted four individual interviews with 2 clinical instructors from PH and another 2 clinical instructors from HN. The major themes that emerged from the analysis described students' and clinical instructors' perceptions of the necessity and efficiency of the switch to VI; the design of the VI and the extent of the clinical/field experience and skills that it offered; confidence in the ability to succeed in this type of internship and confidence about reaching expected goals; academic and moral support from clinical faculty and coordinators and the communication process with faculty and preceptors; and finally, the benefits gained and how employers would view this type of internship.

### Discrepancy

#### The necessity and efficiency of the switch to VI

Most of the students expressed an understanding of the decision to switch to VIs and worry about being in a clinical setting during the pandemic. They also mentioned that their clinical faculty were worried about them contracting the infection from the training site and possibly transmitting the infection to their families.

“Well, like we still wanted to have the experience of clinical practice. However, we were afraid. I was afraid of, you know the situation. So, it was also a relief to me that I will stay at home. I will not, I will not be at risk of having the disease and then passing it to my family, so yeah.” [PH student 3]

A preceptor for HN students also explained that the majority of students understood the need for the shift. The teaching assistant from PH explained that, although students were disappointed that they missed the training in a clinical setting, they understood the safety issues involved.

“The students are mature, it is a safety issue, health issue it is not only what they want, it is a pandemic. They understood it is serious and they did not give us hard time. They understood the shift. They have to work, to create a program and work from home.”

On the other hand, only a few students mentioned that this switch was not necessary since they were aware of the preventive measures they need to follow. These students showed accountability to the profession and felt responsible to support their colleagues in the healthcare system. They also explained that since other health care providers were on the site fighting COVID-19, they should have also joined them. A PH student commented: “It was our area, it was our responsibility. We know how to protect ourselves, and we could have helped more. It is our job to fight this pandemic with other professionals.”

Most students agreed that the college's response and action to the pandemic as well as the switch to online learning and VIs were timely and prompt. A student from HN mentioned: “I think it was a very good response and they did their best to compensate the training while being at home which is hard to do especially when the preceptors are there in the hospital and busy with this situation, but our department response was suitable during COVID19.” Another added, “It was a very quick and adequate response, the best that could have been done with the sudden changes to the entire system.”

### Appropriateness

#### The VI design was not well structured

Students' perceptions regarding the overall design of the VI were generally negative. Only a few PH students were satisfied with some gaining of important research skills as well as online communication and planning skills. The majority of the students mentioned that the PBI did not equip them with the skills they were seeking from the field experience and emphasized that they missed the real hands-on experience they were looking for and missed gaining the skills and competencies needed for a patient educator.

“I think it's not enough just to have a project. Uh, because it's better to go to the site, learn more, explore the working environment. How you deal, interact with patients, yeah. How you will be a professional health educator? This is how we will gain the needed skills and from the training.”

Perspectives of HN students about the appropriateness of the design of the VI reflected several concerns regarding some types of rotations, which they believed were not compatible at all with online delivery. One of the HN students explained how she missed gaining experience with tray line in the food service rotation:

“Also in the food service rotation, I see that we couldn't have the opportunity to see what is happening in the tray line. I was waiting for this rotation, but couldn't have the ability to see what is happening there. So maybe if they could show us pictures, I know that this is against the policy, but this is a new situation. I think the design was good, but if we could shadow a dietitian it would be better.”

Moreover, the majority of HN students mentioned that the VI needed to be more structured and had to allow for more communication with preceptors. They explained that there should be a clear timeline for the daily activities they will be practicing. “I think it needs to be more structured and doing more virtual meetings with the preceptors every day because we benefit a lot when communicating with the preceptors rather than doing the assignments alone,” expressed one of the HN students.

In agreement with students' generally negative perceptions, all clinical instructors mentioned that the overall design of the internship was partially satisfying, partially meeting the students' needs, addressing the course objectives, and achieving the students' learning outcomes:

“This type of internship is another project for them because they also were working on their capstones, they want a different experience. They want real, practical experience. PBI helped improve their research skills, writing skills, communication skills, planning skills, teamwork and collaboration, technology, and online skills. ” [PH Clinical Coordinator]

#### Disappointment with the limited clinical/field experience

While students expressed their understanding of the university's decision, and tried to be supportive of such a policy, the majority expressed their disappointment from missing the opportunity of being in the field and having real practical experience. Most of the PH students were not satisfied with the PBI, because this type of internship did not address their learning needs, nor the objectives of their practicum course. Two HN students explained,

“I don't like the virtual SPP because I feel that like I'm repeating this theoretical semester just doing the assignments as my friend said that there is no time to meet the preceptors and listen to them.”

This was echoed by the clinical instructors who mentioned that students reacted differently when the shift happened, but the majority felt disappointed from missing the opportunity of being in the clinical sites and interacting with other professionals and patients. They explained that students were not satisfied since there was a big difference between being in the field and working from home. The PH clinical coordinator explained:

“We were all challenged; faculty, supervisors, and students. There is a big difference from the real work experience to another public health project through a screen AND from home. Experiential learning is very important for our students, especially for PH students. The PH field is new and challenging in the country, students were looking for this training to apply what they have learned in the classroom and for real hand-on experience.”

### Efficacy

#### Student's confidence in their ability to succeed in this type of internship

Despite their doubt about the appropriateness of the internship design *via* online mode, the majority of the students were confident that they would be able to manage the VI and submit the required assignments, case studies, and working sheets on time. Students from the PH program explained that they were familiar with the steps they need to follow in conducting a PH project, as one student mentioned, “So I could say we were confident; we did the project anyways in a previous course. So, I thought like, yeah we have enough time to be done with the project and all will be fine.” Similarly, HN students expressed that the knowledge they gained from previous practical training along with their good online skills gave them high confidence during their VI.

“I don't doubt that any of our batches don't have the technical skills and knowledge required for this virtual training… The number of skills we used in the practical training was much more than what we needed in the virtual training. [HN student]

PH clinical coordinator confirmed students' confidence in managing the PBI. A clinical instructor from HN added that students were confident in navigating online training and emphasized that students were technology-oriented.

“I think they were confident for a reason is that they were familiar, they have practiced the major steps in planning for a health education program, and I think we can do it and we can work on reviewing the literature, collecting data, and planning…They have been practicing this for years.” [PH Clinical Coordinator]

#### Students lack confidence in reaching expected goals

On the other hand, the majority of students from the CHS mentioned that the VI would not help them reach the internship objectives and gain the needed skills required in a clinical setting. A PH student expressed “I think we were not confident that we will reach our goals, I mean the practicum goals. This is a PH project, not the training we were looking for. Even the instructors themselves; they were not confident about the outcome.” The same negative perceptions were expressed regarding the confidence in own practical skills when a HN student added:

“I do feel confident in the knowledge I have received with regards to the different cases seen at the different rotations and the treatments that are given to them. But, with regards to patient consultation, the practical experience would have definitely helped in building communication skills plus experiencing how to consult different people and personalities.”

Clinical instructors shared the same perceptions regarding the students' lack of gaining some skills during the VI:

“There are many differences, but the main difference is that the lack of ability to assist the students in the development of needed skills such as team-building skills, time management and prioritization in patient care, role-modeling, and evidence-based professional dietitian practice.” [HN Preceptor]

### Principal support

#### Academic and moral support from QU clinical faculty and coordinators

Most of the students held positive perceptions of the support provided by the university faculty and clinical coordinators. Students agreed that the college's response was prompt and that faculty provided an efficient and clear plan. Students mentioned that clinical coordinators and faculty were available and showed a willingness to provide support and instructions at any time. They explained how faculty used different communication channels to assess their needs and listen to their concerns in regards to their internship and even other courses.

“I think the faculty had their agenda constructed, they have limited time, and they were trying to do certain things in a limited time and to accommodate the situation. So, I think what was done in this course and even other courses in the department such as the weekly meetings with the clinical coordinator, were very helpful to direct, assist and support us.” [PH student 5]

Clinical faculty had similar views to those of students regarding academic and emotional support from the college, department, and faculty. They also emphasized they were keen on “Assessing learning needs, providing constructive feedback, and applying effective communication” to students, as indicated by one HN faculty preceptor. The other HN faculty preceptor also indicated that discussions were the main way that preceptors used for clarifying students' concerns regarding any experiences in the rotation.

#### Negative perception of the communication process with preceptors from training sites

The majority of the students were not satisfied with the support and guidance provided by the preceptors from the training sites. They were also not satisfied with the quality and frequency of the communication with these preceptors.

“To reach the goals I think we need to communicate more with the preceptors so we can gain more of their practical experience. Also, to do more tasks other than theoretical assignments, like the counseling.” [Nutrition student 1]

Clinical faculty from PH agreed that students were expecting more support from preceptors and stakeholders:

“We do need external support to give students a little bit of sense of the work in the field. I believe we need to have somebody who gives lectures about what's going in the site. We need guest speakers from the field in addition to the project.” [PH Clinical Coordinator]

### Valence

#### Benefits gained from the VI

Students were asked about their views regarding the long-term benefits of VI. Few PH students mentioned that the PBI improved their research and online communication skills. It also enhanced their online skills in the implementation of health education sessions, data collection, and needs assessment. One PH student mentioned, “I feel the project. Yeah, to write in my CV that we have done research and need assessment. It's I think, it's really good and a positive thing because as a public health educator we should be confident and have these research skills.”

Meanwhile, some students from the nutrition program positively perceived how they learned about navigating online counseling and telehealth and worked under emergency conditions:

“Do online counseling or deal with patients online which could benefit me in my future career if I need to work online. The program prepared me for dealing online with patients and also working under different circumstances and under a global emergency situation in which we experienced this now and if in the future had to work under the same circumstances, we will be able to. [HN student 2]

Clinical instructors shared similar views regarding some of the skills that were provided to students through the VI. The PH clinical coordinator mentioned that students built on their previous skills from the PBI, such as research skills, data collection skills, communication skills (oral and written), collaboration and teamwork, online skills, planning skills, critical thinking, and creativity. A nutrition preceptor mentioned:

“As Food service is an inevitable part of the Dietetics department, the rotation will help the students to apply their knowledge of food systems management and understand the functions of the dietitian in food service and administration. As throughout the rotation they are participating in the supervision of food production, sanitation inspections, menu planning, sensory evaluation and kitchen design.”

#### Students were worried about how employers would perceive this type of internship

Most of the public health students agreed that the PBI would not enrich their resume, nor enhance employability when they apply for jobs in the near future. They were concerned that employers would prefer having an internship in a clinical setting during the pandemic instead of having an online public health project or a project-based internship.

“Not sure how the employers will view this type of internship. Like. I know that it's a new thing, but I don't know how they would accept and view such an internship as they would ask about any previous experience in the field or things like that… I'm not sure how they would see it since it was like 4 credit hours…” [PH student 8]

Students from HN felt a need to compensate for the lost practical experience and suggested:

“After the situation gets better, we could revisit the sites we weren't able to. For the sake of practicality, we could maybe have a choice of choosing 2 or 3 rotations out of all the rotations we have missed, or we could even shorten the time spent in each practical rotation when visiting on-site….”

The PH clinical instructors were also concerned about how employers will view this type of internship and suggested that the department needs to support students and issue certificates explaining this type of internship.

“As I mentioned, the project was realistic they built on their experience from the previous site, this is something to highlight. In addition, they had research skills, writing, literature review, and online presenting skills. Yet, I have a concern, yeah, maybe it won't be taken as a field internship. Maybe we need to support these students with a certificate, saying that this online training was a real training when applying for a job.” [PH teaching assistant]

## Discussion

This study aimed to explore health sciences students' and instructors' experience of an emergent shift to virtual internship and their perceptions of VI replacing on-site experiential learning. Being sensitized to the main constructs of the readiness framework, major themes arose from the analysis reflected on the participants' understanding of the shift to VI, the appropriateness of the overall design of the VI, confidence in one's own abilities to succeed through the VI, efficient communication and instruction, leadership support and technical support, and personal benefits from the VI in the long term.

In general, students had a sufficient understanding of the need to shift to VI and described the decision by the university to move to VI to be prompt and efficient. When exploring student safety during the pandemic, many students perceived that the switch to VI was necessary to maintain their safety, the safety of their families, and that of patients. Such a finding is understandable because our study was done at a time when the pandemic was at its peak, a time when the psychological effects of the pandemic, such as fear and anxiety, were highly seen (29). On the other hand, some students were confident that taking the protective measures could have been enough to maintain their security and that of the patients while not depriving them of having the real clinical training. Moreover, these students expressed a need to be involved in the fight against the pandemic. Similar diversity in the views of students regarding returning to clinical settings was also reported in a study in Brazil (11). However, our results differed from those from a study in Singapore, which assessed the medical students' preferences and showed higher levels of preference for returning to the clinical setting during the COVID-19 pandemic and linked this preference to return to clinical settings with a high level of internal motivation, professional responsibility and little fear that students would pose risk to patients and to the healthcare system (12). Another important theme that emerged from our study indicated that students were disappointed with the limited clinical experience they were offered. This coincides with results from several other studies (8, 10, 30).

The majority of students in our study had negative perceptions about the overall appropriateness of the VI program. A theme from our study indicated that students were not satisfied because the program did not enable them to gain the needed skills and competencies that are usually gained from on-site practice. The students' views about the importance of gaining such competencies are consistent with what was reported in the literature (4–8). Students also felt that the current design of the internship lacked organization and structure. This agrees with results from a study in Brazil (11), conversely, data from KSA show that students were more satisfied and held positive perceptions of the general educational impact of the move to online learning during the COVID-19 pandemic (8). Our findings regarding the perceived inappropriateness in the design of the VI also diverge from several other studies (31–33).

Our study results reflect that students' efficacy depended on certain factors, such as their previous exposures to clinical settings, how much they would be earning essential skills from VIs, and the extent of communication and feedback they were being provided with. Since most of them had the needed online skills and were familiar with clinical settings from their previous rotations, CHS students were confident that they would succeed in this type of internship. Nevertheless, students were not confident that they would reach the expected goals by taking this VI. Such a negative perception was driven by the lack of gaining some of the skills that are essential for clinical practice, such as effective communication with patients and doctors (7), because students were not exposed to real-life interactions with patients, or with the diverse profiles of the healthcare team.

As for their perceptions of support, the majority of students said they received academic and moral support from QU clinical faculty and coordinators. Faculty members provided good quality and frequency of communication with students as well as providing them with timely and constructive feedback. However, there were a few negative student perceptions about the communication process with clinical preceptors assigned from training sites. Only a small number of students were not satisfied with both the quality and the frequency of such communication. It is evident from the literature that such an issue with communication is a major obstacle that usually creates a negative impact on the students' confidence and capability to undertake new challenges and changes (34–36). Our findings regarding student efficacy and the importance of feedback go in accordance with results from other studies (37–39).

Nevertheless, students perceived gaining several benefits from this unique VI. They improved their online skills in data collection and implementation of health education sessions and enhanced their online communication skills. In addition, some of them got exposed to online counseling and telehealth, which according to literature, comprises a valuable asset for the future careers of medical students (40). Finally, students were worried about how employers would perceive this type of internship, which is consistent with findings from another study (41). Previous research reported how SPP enhances employability for nutrition students (42).

Findings from this study provide students' and clinical instructors' insights into the VIs that were being offered as an alternative to the standard onsite clinical training. If students are not ready, they will tend to reject change and face it with negative reactions such as sabotage and absenteeism (36). Just like the case of any organization, one of the main academic institutional tasks and challenges is to ensure the establishment of supportive, cooperative, and trusting relationships that empower both faculty and students and make them more committed (36, 43–45). Although the lack of interaction and lack of clinical experience tended to be the main perceived concerns students expressed toward shifting to VIs, the literature suggests that there is room to overcome such barriers by integration of new learning methods such as simulated patients, case based learning and telehealth (9). In that context, challenges to student readiness, which are due to the nature of some rotations, can be faced by tailoring the rotation in a way that ensures more benefit and confidence to students.

The findings of our study set a base for future research that can suggest what improvements should be implemented in the design of the VI program, and evaluate the impact of such improvements. In that sense, it would be important to consider the introduction of new interactive learning techniques that can enhance students' clinical skills and hence provide more benefits from the VI. Moreover, this study shed the light on several student and faculty needs that should be addressed to optimize the students' experience of VI. There will be an urgent need for training of preceptors, specifically in relation to improving their skills of communication and providing feedback during VIs.

Applying qualitative research methods helped us recruit rich cases and understand the experience in depth. In order to enhance the credibility of our findings, we applied triangulation is different ways: data collection through interviews and field notes, and independent data analysis by three members of the research team. On the other hand, one of the principal limitations of the study is the inability to have the themes validated by the study participants due to time constraints. The study assessed students' readiness from the perspective of members and students from only one health college of QU. However, to enrich the understanding and provide broader views of the VI it would be important to assess the perspectives of students and members of other health colleges in QU and those in other universities in Qatar.

## Conclusion

Results from this study highlighted health sciences students' experience in switching to VI during the COVID-19 pandemic in Qatar. Our study revealed that students held several negative perceptions about the different aspects of the change, and hence their readiness for shifting to VI was generally low. However, the study suggested other positive views including the prompt and efficient decision by the university to move to VI, good moral support from faculty and preceptors as well as several perceived benefits of this type of training. The finding would support planning for efficient VI for health sciences students.

## Data availability statement

The raw data supporting the conclusions of this article will be made available by the authors, without undue reservation.

## Ethics statement

The studies involving human participants were reviewed and approved by Qatar University Institutional Review Board (QUERG-CHS-2020-1). The patients/participants provided their written informed consent to participate in this study.

## Author contributions

HB, HA, XD, AE-A, and AA-M: conceptualization of the study, designing the interview guides, and critical review of the manuscript. JM: data collection and write-up of the manuscript. RS and MD: write-up of the manuscript. GA-J: conceptualization of the study, designing the interview guides, data collection, data analyses, write-up of the manuscript, critical review of the manuscript, journal submission, and response to reviewer comments. All authors contributed to the article and approved the submitted version.

## Funding

This study was funded by a Qatar University Emergency Response Grant (QUERG-CHS-2020-1).

## Conflict of interest

The authors declare that the research was conducted in the absence of any commercial or financial relationships that could be construed as a potential conflict of interest.

## Publisher's note

All claims expressed in this article are solely those of the authors and do not necessarily represent those of their affiliated organizations, or those of the publisher, the editors and the reviewers. Any product that may be evaluated in this article, or claim that may be made by its manufacturer, is not guaranteed or endorsed by the publisher.

## Abbreviations

CHS: College of Health Sciences
HN: Human Nutrition
HMC: Hamad Medical Corporation
KSA: Kingdom of Saudi Arabia
PH: Public Health
PBI: Project-based internship
QU: Qatar University
QU Health: Qatar University Health
VI: Virtual Internship
QDA: Qatar Diabetes Association
USA: United States of America.
